# Relationship between Quality of Life, Relationship Beliefs and Attribution Style in Infertile Couples 

**DOI:** 10.22074/ijfs.2018.5221

**Published:** 2018-03-18

**Authors:** Behnaz Navid, Maryam Mohammadi, Saman Maroufizadeh, Payam Amini, Zahra Shirin, Reza Omani-Samani

**Affiliations:** Department of Epidemiology and Reproductive Health, Reproductive Epidemiology Research Center, Royan Institute for Reproductive Biomedicine, ACECR, Tehran, Iran

**Keywords:** Assisted Reproduction Technique, Attribution Style, Infertility, Quality of Life, Relationship Beliefs

## Abstract

**Background:**

Many infertile couples experience psychological distress and suffer from impaired quality of life. Gen- erally, when couples are dealing with uncontrolled events such as infertility, it is important to manage it well and to use the suitable coping style; so this can represent an example of attribution style. The purpose of this study is to investigate the quality of life, relationship beliefs and attribution style in infertile couples.

**Materials and Methods:**

This cross-sectional study consisted of 50 infertile couples, who were at least 18 years of age and could read and write in Persian. Participants provided demographic and general characteristics and completed the quality of life (SF-12), relationship belief inventory (RBI) and attribution style (ASQ) forms. Data was analyzed by the paired t test, Pearson correlation tests and multiple linear regression analysis, using SPSS version 22 statistical software.

**Results:**

Overall, 50 infertile couples participated in our study. The males had a significantly higher score for quality of life compared to the females (P=0.019). In RBI subscales except “Disagreement is Destructive” all others signifi- cantly higher in wives than husbands. All subscales of RBI had a negative correlation with the quality of life. The quality of life had a significant correlation with positive internal (r=0.213, P=0.033). The adjusted regression model showed that the quality of life for males was higher than in females (β=-3.098, P=0.024).

**Conclusion:**

The current data indicate that in infertile couples, the husbands have a higher quality of life in comparison to their wives. Also, all subscales of relationship beliefs have a negative correlation with the quality of life, but in at- tribution style, just internal attribution style for positive events is associated with the quality of life. In general, there is a correlation between relationship beliefs and the quality of life in infertile couples.

## Introduction

It is known well Having children has always been considered as a major sociocultural value in many societies (1). Prevalence of infertility is currently estimated at 9% worldwide (2), and affects approximately 2.5% of the Iranian population (3). Infertility could lead to serious emotional problems and psychological stress (4-7). It potentially has negative impact on marital happiness, sexual satisfaction and the general quality of life (8-11). Generally, the quality of life is affected by a person’s physical health, psychological condition, social constraints and personal belief (12, 13), the latter being a key determinant in the quality of the relationship between spouses (14).

Rational relationship is the most common problem explained by dissatisfied couples. Many mental health issues occur if spouses not to speak about or meet their needs (15). A number of studies have shown that there is a negative association between irrational believes and marital satisfaction (16-18). When infertile couples are dealing with this uncontrolled event, it is very important how to evaluate it in order to use a suitable coping style; so this can represent an example of attribution style. Peterson and Seligman (19) argue the causal styles in three main attribution dimensions as (internality versus externality), (globality versus specificity) and (stability versus instability).

Attribution style theory provides a framework for understanding the causal statements that individuals explain their own behavior as well as the behavior of others (20). Based on this theory, when individuals tend to interpret negative events to internality (self), globality and stability, they are more prone to experience mental problems (21). Some studies suggest that individuals with depression tend to use internal attributions for negative events (20, 22).

Therefore, it is import to know how relationship beliefs and attributionstyles can impact on one’s quality of life. To our knowledge, this represents the first study to exam. ine this relationship in infertile couples. The aim of this study is to investigate the connection between the quality of life based on relationship beliefs and attribution styles in infertile couples.

## Materials and Methods

A cross-sectional study was conducted on infertile couples who referred to the Royan Institute, a specialty infertility clinic in Tehran, the capital of Iran, between January and February 2014. Correlation sample size formula with a 0.4 correlation between variables from same study and was used to find an optimal sample size. The sample size with a confidence interval of 95%, power of 80%, and with a significance level of 0.05, was estimated to be 50 couples (50 males and 50 females). The sampling method was available sampling. The inclusion criteria were being 18 years or older, having a history of infertility, and being able to read and write in Persian.

The study was approved by the Ethics Committee of Royan Institute. Aims of the study and the confidentiality of the data were clearly explained for all participants. Eligible individuals were also assured that acceptance or refusal to participate in the research had no influence on their treatment procedures. Voluntarily filling the questionnaire was considered as consent.

### Instruments

#### Demographic

The demographic questionnaire include age (years), educational levels (under diploma,under diploma and academic), type of infertility (male factor, female factor, unknown, both), duration of infertility (year), duration of marriage (year).

#### 12-Item Short-Form Survey (SF-12)

The quality of life (the short form health survey) SF- 12 used in our study is a Persian version of the SF-36 instrument. The SF-12 was developed in 1996 by Ware et al. (23) in English as part of the International Quality of Life Project Assessment, a shorter alternative to the SF-36, and was translated subsequently into Persian and other languages. In the Persian version of SF-12 validated in 2007 by Montazeri et al. (24), Cronbach’s alpha coefficient (to test reliability) was 0.85, and intraclass correlation coefficient (ICC) was 0.90. Cronbach’s alpha coefficient in this study was 0.77. The SF-12 questionnaire was used as a common tool for evaluating the quality of life of infertile couples (25). The SF-12 is comprised of eight subscales: physical functioning, physical role, bodily pain, general health, vitality, social functioning, emotional role, and mental health. These were summarized into two scales: a physical component score (PCS) and a mental component score (MCS), in accordance with the guidelines for the SF-12 instrument. Both scores ranged between 12 and 48, with a higher score indicating better health. These SF-12-based summaries have been shown to reproduce accurately both PCS and MCS, which have been derived from the full SF-36 (26).

#### Relationship Belief Inventory

Relationship Belief Inventory (RBI) questionnaire was published in 1981 by Eidelson and Epstein (26). It is a 40- item self-report instrument and contains disagreement is destructive (D), partners cannot change (C), and mindreading is expected (M), sexual perfectionism (S) and sexes are different (MF). By summing questions for each subscales, the score of each subscale was calculated. In the Persian version of RBI, which was validate by Mazaheri and Pooretemad in 2001 (27), Cronbach’s alpha coefficient was 0.75 and ICC was 0.78. Higher scores indicate a worse relationship belief. Cronbach’s alpha coefficient of total score in this study was 0.80 and Cronbach’s alpha coefficients were 0.60, 0.75, 0.62, 0.67, 0.71 for D, M, C, S and MF respectively.

#### Attribution Style Questionnaire

The Attribution Style Questionnaire (ASQ) was built in 1982 by Peterson et al. (28). This questionnaire contains hypothetical situations split equally into positive AS for (a) good affiliative and (b) good achievement-related situations, and negative AS for (a) bad affiliative and (b) bad achievement-related situations. Respondents rated each causal attribution on three 7-point scales: i. Internality- externality, ii. Stability-instability, and iii. Globality- specificity. Internality, stability, and globality are usually simply summed to derive composite positive attribution style and composite negative attribution style. Cronbach’s alpha coefficient of total score in this study was 0.72 and cronbach’s alpha coefficients were 0.62, 0.71 and 0.76 for internality, stability, and globality respectively.

### Statistical analysis

Statistical analysis were performed using the Statistical Package for the Social Sciences; SPSS V.22.0 for Windows (IBM SPSS V.22.0.0). Continuous variables were expressed as mean ± SD and categorical variables as frequencies (%). Normality of the variables was checked with Kolmogorov-Smirnov Test. Pearson correlation coefficient was used to examine the relationship between study variables, and paired t test was used to evaluate the difference between male and female data (wives and husbands). Finally, multiple linear regression analysis was performed by controlling confounders. P<0.05 was considered statistically significant. 

## Results

### Participants characteristics

In this study, 50 infertile couples consisting of 50 (50%) men and 50 (50%) women participated. The mean age was 33.30 ± 5.13 for males and 28.94 ± 5.26 for females (P<0.001). Amongst the participants 35 individuals (35%) had an academic education. According to the cause of infertility, 49 couples (49%) involved in male factor infertility, 8 (8%) female factor, 7 (7%) both and 36 (36%) unknown. The mean duration of infertility was 4.7 ± 3.84 years and the mean duration of marriage was 6.8 ± 3.89 years in the participants (Table 1).

**Table 1:** Demographic characteristics of the infertile couples


		Male Mean ± SD or n (%)	Female Mean ± SD or n (%)	P value

**Age (Y)**		33.30 ± 5.13	28.94 ± 5.26	<0.001
**Education**				0.017
	** Under diploma/ Diploma**	33 (66)	33 (66)	
	** Academic**	17 (34)	13 (34)	
**Cause of infertility**				
	** Male factor**	49 (49)		
	** Female factor**	8 (8)		
	** Both**	7 (7)		
	** Unknown**	36 (36)		
**Duration infertility**		4.7 ± 3.84		
**Duration of marriage**		6.8 ± 3.89		


### Univariate analysis

The mean score for the quality of life was significantly different between wives and husbands and it was higher in husbands than wives (P=0.019, Table 2). 

Between the factors of RBI questionnaire, Mindreading is Expected (M), Partners Cannot Change (C), Sexual Perfectionism (S) and Sexes Are Different (MF) were significantly different between wives and husbands and they were all higher in wives than in their husbands (P=0.008, 0.001, 0.035 and 0.010 respectively). The husbands had a relatively higher score of Disagreement is Destructive (D) compared to their wives, but the difference was not significant (P=0.152). Overall, the wives expressed a higher mean total RBI compared to their husbands and the overall difference was significant (P=0.002). In the attribution style questionnaire, the scores for negative internality and cynical attributions were not significantly different among males and females have a higher mean score in husbands compared to their wives but not significant. Also, there was no significant difference between males and females with regard to casual attribution categories. 

**Table 2:** SF-12, RBI and ASQ subscales between couples


		Male Mean ± SD	Female Mean ± SD	P value^*^

**SF-12**		38.02 ± 5.12	34.64 ± 5.45	0.019^*^
**RBI subscales**				
	** Disagreement is destructive**	19.90 ± 4.19	18.74 ± 4.60	0.152
	** Mindreading is expected**	21.90 ± 3.89	24.16 ± 5.28	0.008
	** Partners cannot change**	18.44 ± 6.24	21.90 ± 6.09	0.001
	** Sexual perfectionism**	22.68 ± 7.79	25.48 ± 6.61	0.035
	** Sexes are different**	17.48 ± 7.55	20.44 ± 5.24	0.010
	** Total**	100.40 ± 21.94	110.72 ± 20.31	0.002
**ASQ subscales**				
	** Negative internal**	4.18 ± 1.15	3.79 ± 1.06	0.448
	** Positive internal**	4.75 ± 1.17	5.08 ± 1.01	0.702
	** Negative stability**	3.81 ± 0.81	3.89 ± 0.82	0.179
	** Positive stability**	4.78 ± 0.99	4.87 ± 1.04	0.408
	** Negative public**	3.82 ± 1.26	4.04 ± 0.89	0.200
	** Positive public**	4.58 ± 1.34	4.79 ± 1.07	0.573
	** Cynical attribution**	11.81 ± 1.92	11.72 ± 1.72	0.244
	** Optimistically attribution**	14.12 ± 2.98	14.75 ± 2.60	0.263


RBI; Relationship Belief Inventory, ASQ: Attribution Style Questionnaire, SF-12: 12-Item Short Form Survey, and *; Paired t test.

**Table 3:** The relationship of SF-12 with RBI and ASQ subscales


	1	2	3	4	5	6	7	8	9

1. SF-12	1	0.380^*^	0.210^*^	0.119	0.101	0.206^*^	0.386^**^		
2. Disagreement is destructive	0.380^**^	1	0.319^**^	-0.012	0.191	0.202^*^	0.663^**^		
3. Mindreading is expected	0.210^**^	0.319^**^	1	-0.151	0.082	0.097	0.490^**^		
4. Partners cannot change	0.119	-0.012	-0.151	1	0.195	0.257^*^	0.456^**^		
5. Sexual perfectionism	0.101	0.191	0.082	0.195	1	-0.027	0.487^**^		
6. Sexes are different	0.206^*^	0.202^*^	0.097	0.257^*^	-0.027	1	0.600^**^		
7. Total	0.386^**^	0.663^**^	0.490^**^	0.456^**^	0.487^*^	0.600^**^	1		
									
1. SF-12	1	0.061	0.213^*^	-0.166	0.110	-0.029	0.118	-0.054	0.175
2. Negative internal	0.061	1	0.100	0.144	-0.127	-0.031	-0.135	0.661	-0.065
3. Positive internal	0.213^*^	0.100	1	-0.226	0.561^**^	0.265^*^	0.644^**^	0.120	0.876^**^
4. Negative stability	-0.166	0.144	-0.226^*^	1	-0.017	0.010	-0.099	0.540	-0.138
5. Positive stability	0.110	-0.127	0.561^**^	-0.017	1	0.125	0.466^**^	-0.009	0.786^**^
6. Negative public	-0.029	-0.031	0.265^*^	0.010	0.128	1	0.526	0.586^**^	0.378^*^
7. Positive public	0.118	-0.135	0.644^**^	-0.099	0.466^**^	0.526^**^	1	0.189	0.855^**^
8. Cynical attribution	-0.054	0.661^**^	0.120	0.540^**^	-0.009	0.586^**^	0.189	1	0.126
9. Optimistically attribution	0.175	-0.065	0.876^**^	-0.138	0.786^**^	0.378^**^	0.855^**^	0.126	1


RBI; Relationship Belief Inventory, ASQ; Attribution Style Questionnaire, SF-12; 12-Item Short Form Survey, r; Pearson correlation coefficient, *; P<0.05, and **; P<0.001

### Correlations among study variables

Additionally, bivariate correlations were conducted among subscales of two questionnaires (RBI and ASQ) with the score of quality of life (SF-12), as shown in Table 3. In subscales of the RBI questionnaire, D, M, C, S, MF and a total of RBI were positively correlated with the total score of quality of life, but only D, M, S and the total score of RBI had a significant correlation. In addition, positive internality was directly and significantly correlated with total score of SF-12 (P=0.033).

### Multiple linear regression analysis

For the quality of life, the enter method was used as a covariate selection method, in step 1, demographics and subscales of RBI ad ASQ entered to model, sex and “Disagreement is Destructive” were significantly related to quality of life (ß=-3.098, P=0.024 and 0.254, P=0.023 respectively). When gender, education, duration of marriage, duration of infertility, subscales of RBI and subscales of ASQ were in the quality of life model, the model adjusted R2 was equal to 0.134. On the other hand, variance inflation factor (VIF) and tolerance of variables showed the model does not have collinearly. In step 2, sex and “Disagreement is Destructive” entered to model “Disagreement is Destructive” was positively correlated with the quality of life (ß=0.319, P=0.001) and gender was negatively correlated with the quality of life. (ß=-2.499, P=0.001) When sex and “Disagreement is Destructive” were in model, there was an improvement in the model (adjusted R2=0.176, P<0.001, Table 4).

**Table 4:** The results of hierarchical multiple linear regressions, including factors related to the quality of life


	Quality of life			
	P	Beta	SE	B

Step 1:				
Sex (female vs. male)	-3.098	7.986	-0.282	0.024
Education (educated vs. under diploma/diploma)	1.260	1.225	1.225	0.307
Duration of marriage	-0.101	0.303	-0.071	0.739
Duration of infertility	0.146	0.305	0.101	0.633
Age	-0.133	0.152	-0.135	0.385
Disagreement is destructive	0.254	0.110	0.257	0.023
Mindreading is expected	0.121	0.129	0.102	0.349
Partners cannot change	0.048	0.130	0.041	0.714
Sexual perfectionism	0.055	0.133	0.044	0.680
Sexes are different	0.055	0.113	0.054	0.631
Negative internal	0.058	0.538	0.012	0.914
Positive internal	-0.351	1.532	-0.070	0.819
Negative stability	0.125	0.751	0.018	0.869
Positive stability	-0.797	1.049	-0.147	0.450
Negative public	-0.402	0.627	-0.079	0.523
Positive public	0.539	0.458	0.118	0.242
Cynical attribution	-0.165	0.307	-0.054	0.593


## Discussion

Our results revealed that husbands have a higher quality of life in comparison to their wives and generally males have a higher quality of life than females. In the Iranian culture, the paternalistic beliefs for fertility and the lack of social and economic supports for many women are some factors that may amplify the psychological problems of infertile women. Based on cultural conditions, such women are more likely to be under mental and emotional pressures (29). The results presented here are in line with previous studies that had shown that males reported a higher quality of life than females (25, 30-32). 

Our study about factors related to relationship beliefs showed that the wives expressed a higher mean score in relationship beliefs than their husbands, meanings that wives had unreasonable relationship beliefs compared to their husbands. Also, our results indicated that relationship beliefs negatively correlate with the quality of life except for “Disagreement is Destructive” subscale, which is positively correlated with the quality of life. Many couples believe that if they oppose to their spouses, it is destructive and makes them more compatible with marital life, and as a result, their quality of life goes up. Irrational beliefs were positively related to different types of distress, such as general distress, anxiety, depression and anger (33).

A previous study reported that irrational beliefs relate to health-related quality of life (34, 35). The result of this study is nearly in line with another previous study in marital satisfaction, which reported a correlation between marital dissatisfaction and unreasonable relationship beliefs (15, 36). The results of this study also revealed that only one n internal attribution style for positive events was associated with the quality of life and the remaining dimensions of attribution style (ASQ total score, global and stable causal attributions of negative and positive events) did not display a significant relationship with the quality of life. Goli et. al. (37) have examined the roles of locus of control and attributional style in coping strategies and the quality of life between patients with cancer. The results from their study approved a positive correlation among optimistic attributional style and the quality of life.

A study reported that attribution style was significantly associated with psychopathology scores as the patients that tend to internalize, showed greater overall psychopathology (20). Also, a number of studies have shown that individuals with depression tend to excessively use internal attributions for negative events (38, 39). Therefore, attribution styles are useful for social cognitive predictors and psychosis improvement. Since the relationship beliefs have an essential role in satisfaction or dissatisfaction of life, we can change the attribution styles to improve psychosis disorders. It means that changing these beliefs can help couples to have longer and happier marital lives. 

Finally, there is a need to educate infertile couples, so that they have realistic expectations, successful relationships and good quality of life. These could be provided with the help of the couple’s therapists, family life educators, or psychologists. The findings of this study enable us to design and analyze future studies, which will have larger sample sizes from multiple infertility centers, with improvements.

## Conclusion

The results of the current study indicate that in infertile couples, men have a higher quality of life in comparison to their wives. Also, all subscales of relationship beliefs have a negative correlation with the quality of life. However, in attribution style only internal attribution style for positive events was associated with the quality of life. In general, there is a correlation between relationship beliefs and the quality of life in infertile couples.
